# Fast, simple and highly specific molecular detection of *Vibrio alginolyticus* pathogenic strains using a visualized isothermal amplification method

**DOI:** 10.1186/s12917-020-02297-4

**Published:** 2020-03-04

**Authors:** Yu Dong, Panpan Zhao, Li Chen, Huahua Wu, Xinxin Si, Xin Shen, Hui Shen, Yi Qiao, Shanyuan Zhu, Qiong Chen, Weiwei Jia, Jingquan Dong, Juan Li, Song Gao

**Affiliations:** 1 Jiangsu Key Laboratory of Marine Biological Resources and Environment, Jiangsu Key Laboratory of Marine Pharmaceutical Compound Screening, Co-Innovation Center of Jiangsu Marine Bio-industry Technology, Jiangsu Ocean University, Lianyungang, 222005 China; 2 Key Laboratory of Zoonosis Research by Ministry of Education, College of Veterinary Medicine, Jilin University, Changchun, 130062 China; 3 Jiangsu Institute of Oceanology and Marine Fisheries, Nantong, 226007 China; 4 Jiangsu Agri-animal Husbandry Vocational College, Taizhou, 225300 China; 5 Wuhan Institute for Food and Cosmetic Control, Wuhan, 430000 China; 6 School of Pharmacy, Jiangsu Ocean University, Lianyungang, 222005 China

**Keywords:** *Vibrio alginolyticus*, Molecular detection, Isothermal amplification, Recombinase polymerase amplification, Lateral flow dipstick, Specific

## Abstract

**Background:**

*Vibrio alginolyticus* is an important pathogen that has to be closely monitored and controlled in the mariculture industry because of its strong pathogenicity, quick onset after infection and high mortality rate in aquatic animals. Fast, simple and specific methods are needed for on-site detection to effectively control outbreaks and prevent economic losses. The detection specificity towards the pathogenic strains has to be emphasized to facilitate pointed treatment and prevention. Polymerase chain reaction (PCR)-based molecular approaches have been developed, but their application is limited due to the requirement of complicated thermal cycling machines and trained personnel.

**Results:**

A fast, simple and highly specific detection method for *V. alginolyticus* pathogenic strains was established based on isothermal recombinase polymerase amplification (RPA) and lateral flow dipsticks (LFD). The method targeted the virulence gene *toxR*, which is reported to have good coverage for *V. alginolyticus* pathogenic strains. To ensure the specificity of the method, the primer-probe set of the RPA system was carefully designed to recognize regions in the *toxR* gene that diverge in different *Vibrio* species but are conserved in *V. alginolyticus* pathogenic strains. The primer-probe set was determined after a systematic screening of amplification performance, primer-dimer formation and false positive signals. The RPA-LFD method was confirmed to have high specificity for *V. alginolyticus* pathogenic strains without any cross reaction with other *Vibrio* species or other pathogenic bacteria and was able to detect as little as 1 colony forming unit (CFU) per reaction without DNA purification, or 170 fg of genomic DNA, or 6.25 × 10^3^ CFU/25 g in spiked shrimp without any enrichment. The method finishes detection within 30 min at temperatures between 35 °C and 45 °C, and the visual signal on the dipstick can be directly read by the naked eye. In an application simulation, randomly spiked shrimp homogenate samples were 100% accurately detected.

**Conclusions:**

The RPA-LFD method developed in this study is fast, simple, highly specific and does not require complicated equipment. This method is applicable for on-site detection of *V. alginolyticus* pathogenic strains for the mariculture industry.

## Background

*Vibrio alginolyticus* belongs to the *Vibrio* genus in the Vibrionaceae family. *V*. *alginolyticus* is a Gram-negative, rod-shaped, flagellar bacterium that has halophilic features and widely exists in ocean and estuarine areas [1–3]. *V. alginolyticus* is considered one of the most harmful *Vibrio* species and is pathogenic to both humans and aquatic animals [2, 4]. In humans, soft tissues, the ear, and superficial wounds are easily invaded by *V*. *alginolyticus* when exposed to contaminated seawater [4]. Clinical symptoms include chronic diarrhea, otitis and wound infection [5, 6]. For aquatic animals, *V*. *alginolyticus* causes a variety of diseases, such as septicemia of *Sparus aurata* [7, 8], exophthalmia and corneal opaqueness of *Epinephelus* spp. [9], melanosis of *Rachycentron canadum* [10], white spot syndrome of *Penaeus vannamei* [11], necrosis of *Macrobrachium rosenbergii* larvae [12], and massive mortality of *Tapes decussatus* [13, 14]. Control of *V. alginolyticus* has to be effective in the mariculture industry due to its strong pathogenicity, quick onset after infection and high mortality rate [15].

As a *Vibrio* species, *V*. *alginolyticus* shares many similarities with other members of this family, including its morphology, surface antigens, virulence factors, genome sequences, and early symptoms of infection [16–18]. These similarities make specific detection of *V*. *alginolyticus* and distinguishing it from other *Vibrio* species difficult. Conventional culturing, immunodetection and symptom judgement can be used to identify a *Vibrio* infection but not at the level of species specificity [19–21]. It should be noted that not only is the severity of infection caused by different *Vibrio* species different but the treatment methods have different outcomes [22]. As one of the most virulent *Vibrio* species in the mariculture industry, *V*. *alginolyticus* has to be specifically detected for pointed treatment and prevention [23, 24].

Polymerase chain reaction (PCR)-based molecular detection methods have been developed for the specific detection of *V*. *alginolyticus* because they can select and target specific sequences on the *V*. *alginolyticus* genome [25]. Virulence genes, including *tlh* (thermolabile hemolysin), *tdh* (thermostable direct hemolysin), *toxR* (cholera toxin transcriptional activator), *sto* (heat stable heat-stable enterotoxin), *ctxA* (cholera toxin A subunit) and *vpi* (*V. cholerae* pathogenicity island), are the most frequent targets used in PCR-based detection of *V*. *alginolyticus* [26–28]. Due to the complicated mechanism of pathogenicity of *V*. *alginolyticus*, a pathogenic strain may not have all of the virulence genes. To date, only the *toxR* gene has shown good coverage for all pathogenic strains [29, 30].

Although PCR-based specific detection methods for *V*. *alginolyticus* targeting the *toxR* gene are promising, they cannot fulfill the current requirement for on-site detection by the mariculture industry because they require complicated thermal cycling devices and trained personnel [31]. Recombinase polymerase amplification (RPA) is an isothermal in vitro nucleic acid amplification technology that is fast, simple and specific [32]. Combining recombinase and polymerase activities, RPA opens DNA strands and amplifies DNA targets isothermally. Chemical labeling enables end-point reading of the amplification product as a colored signal from gold nanoparticles (AuNPs) by the naked eye on lateral flow dipsticks (LFDs) [32]. The RPA-LFD method has been applied for the detection of many pathogens, including goose parvovirus [33], *Burkholderia mallei* [34], *Trichinella* spp. [35], *Mycoplasma bovis* [36], *Candidatus* Liberibacter asiaticus [37], *Pasteurella multocida* [38] and *Phytophthora soja* [39].

In this study, we developed an RPA-LFD method that highly specifically detected *V*. *alginolyticus* pathogenic strains. The virulence gene *toxR* was targeted, and the primer-probe set for the RPA reaction was designed to specifically recognize *V*. *alginolyticus* pathogenic strains but not other *Vibrio* species or other pathogenic bacteria. The RPA-LFD method finished detection within 30 min under an isothermal temperature between 35 °C and 45 °C. The rapidness, simplicity and high specificity of this method make it well suited for on-site detection of *V*. *alginolyticus* pathogenic strains.

## Results

### RPA-LFD method for the visual detection of *V. alginolyticus*

**Fig. 1:**
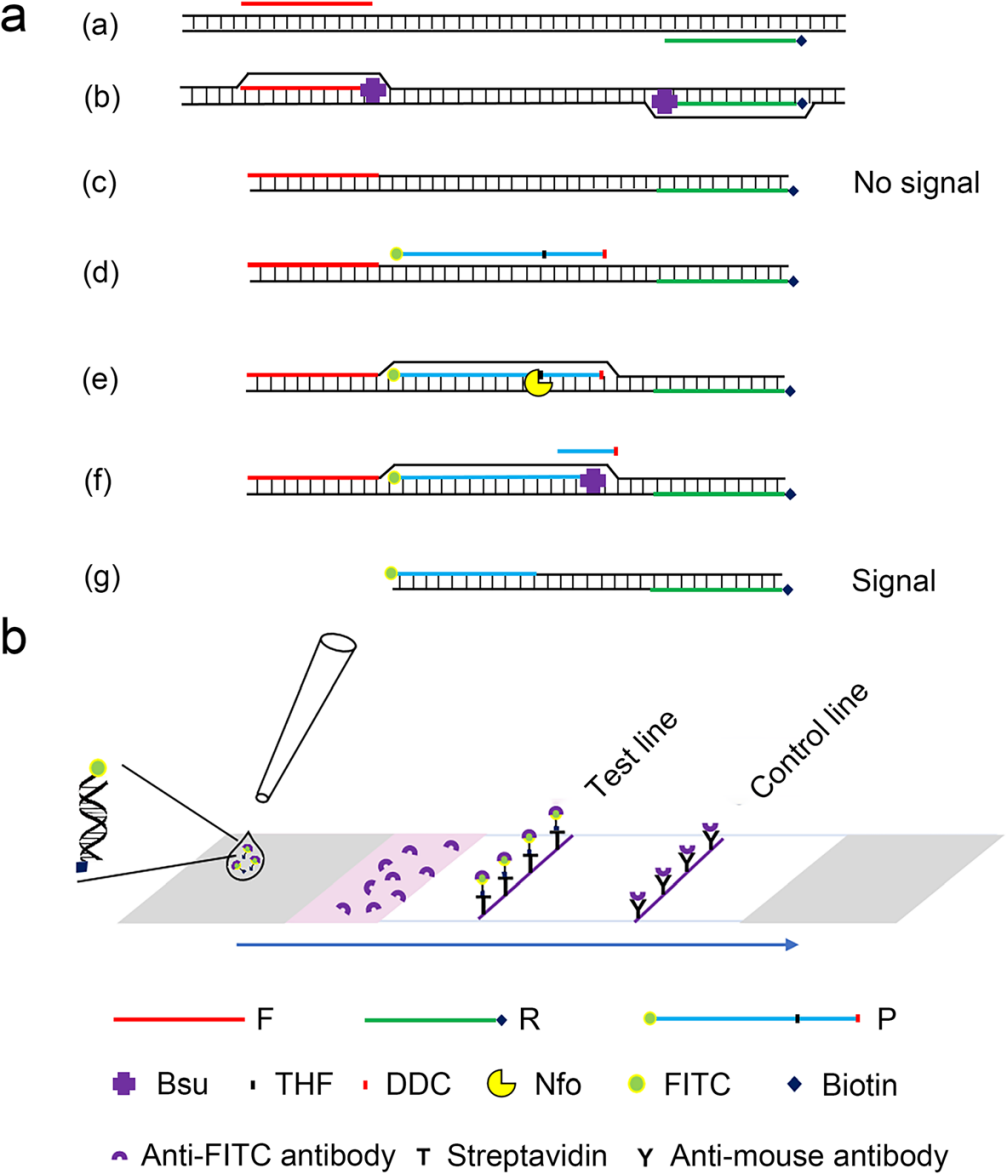
Schematic diagram of the RPA-LFD method. (**a**) Principle of RPA amplification. DNA strands are presented as horizontal lines, and base pairings are indicated as short vertical lines between the DNA strands. The forward primer (F), reverse primer (R), probe (P), Nfo, Bsu and modifications on DNA are indicated with different shapes and colors. The legends are given at the bottom of the image. (**b**) Schematic diagram of the working principle of the lateral flow dipstick. The sample pad is indicated by the gray parallelogram on the left, the absorbent pad is indicated by the gray parallelogram on the right, and the conjugate pad is shown in pink. Liquid migration direction is indicated by an arrow. Molecules could be trapped by the materials on the test line, and the control line is indicated by different shapes. Shapes and their representative molecules are listed at the bottom of the image

The molecular detection of *V*. *alginolyticus* is based on recombinase polymerase amplification of a specific DNA fragment on its genome and visualization of the amplification product on a lateral flow dipstick (Fig. 1). Amplification starts with the pairing of the forward (F) and reverse (R) primers to the amplification target on the genome (Fig. 1a, step a). With the help of a recombinase, the double strand opens, and extension from the 3′ ends of the primers is conducted by *Bsu* DNA polymerase (Fig. 1a, step b). This method does not require thermal cycling, and exponential amplification is performed isothermally (Fig. 1a, step c). A specially designed probe (P) binds to the amplification product and facilitates strand displacement (Fig. 1a, steps d-g). The probe has a dideoxycytidine (DDC) at the 3′ end that blocks strand extension and a THF site in the middle that is cleaved by the Nfo enzyme after pairing. Because the Nfo enzyme only cleaves when the flanking bases of the THF site are paired and the polymerase only extends the strand when the C3-spacer is cleaved, using this specially designed probe significantly increases the amplification specificity [32]. The reverse primer and probe were modified with biotin and fluorescein isothiocyanate (FITC) at the 5′ ends, respectively. Only amplification products that have both labels can produce a signal on the lateral flow dipstick.

When the amplification product is spotted onto the lateral flow dipstick, it migrates through an area that is preloaded with an anti-FITC antibody functionalized with AuNPs and is then trapped by biotin-streptavidin affinity at the test line where streptavidin is coated (Fig. 1b). Because of the FITC label, the amplification product binds to the anti-FITC antibody when migrating through the dipstick and produces a red color at the test line where it is trapped. The control line coated with an anti-mouse antibody is located farther from the spotting position than the test line. The anti-FITC antibody produced from mice can bind to the anti-mouse antibody when it reaches the control line and produces a red color to validate the dipstick test. With the use of this lateral flow dipstick, the specific amplification product of *V*. *alginolyticus* DNA is visually detected by observing the red lines on the dipstick.

### Design of the primer-probe set for specific detection of *V. alginolyticus* pathogenic strains

**Fig. 2:**
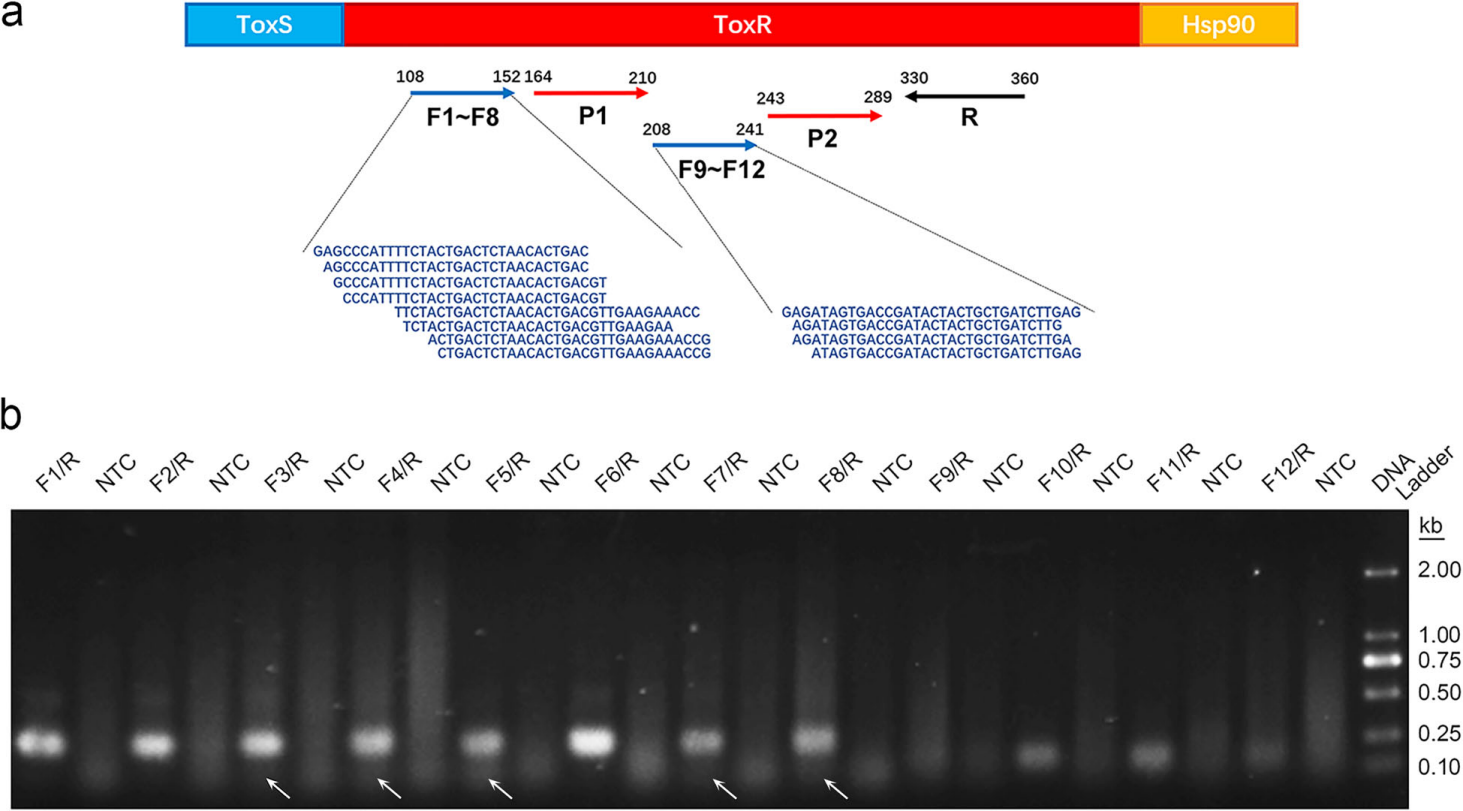
Relative positions of the primer/probe sequences and the initial screening of the primer pairs. (**a**) The upper horizontal line represents the toxR gene and its relative position on the genome. The blue arrows indicate the forward primer positions and the amplification direction. The red arrows indicate the probe positions and the amplification direction. The black arrow indicates the reverse primer position and the amplification direction. The forward primer sequences are written under their respective positions. (**b**) Agarose gel image showing the amplification results for the 12 primer pairs. The primer pair name is indicated at the top of each lane. The NTC lane immediately after is the no-template control of the respective primer pair. The band sizes of the DNA ladder are shown on the right. The white arrows indicate primer dimer bands

The virulence expression regulatory gene *toxR* of *V*. *alginolyticus* was selected as the representative gene for all pathogenic strains [30]. To specifically target *V*. *alginolyticus* but not other *Vibrio* species, the sequence of the *toxR* gene from *V*. *alginolyticus* was compared with those from 7 other *Vibrio* species, including *V. parahaemolyticus*, *V*. *anguillarum*, *V*. *vulnificus*, *V. harveyi*, *V*. *mediterranei*, *V*. *shilonii* and *V*. *cholera* (Figure S1). Regions 90 to 316 and 329–362 (base numbers of the *toxR* gene from *V*. *alginolyticus*) were found to have good sequence diversity. To ensure that the primer-probe set was able to detect all pathogenic strains of *V*. *alginolyticus*, the sequences of the *toxR* gene of all these strains were aligned (Figure S2). Region 50 to 518 (the base numbers of the *toxR* gene from *V*. *alginolyticus*) was found to have good conservation. Combining the sequence diversity and conservation information, 5 candidate regions were selected for the design of the primer-probe set (Fig. 2a). To avoid the formation of primer-probe complexes, a criterion was used such that in a given primer-probe set, the primers and probe needed to have less than 3 consecutive bases (and less than 1 if located at the 3′ end) that paired with each other. Possible sequences of 12 forward primers (divided into 2 groups), 1 reverse primer and 2 probes were obtained (Fig. 2a and Table 1).

**Table 1:** Primers and probes used in this study

Name	Sequence (5′ to 3′)	Amplicon size
F1	5′-GAGCCCATTTTCTACTGACTCTAACACTGAC	252
F2	5′-AGCCCATTTTCTACTGACTCTAACACTGAC	251
F3	5′-GCCCATTTTCTACTGACTCTAACACTGACGT	250
F4	5′-CCCATTTTCTACTGACTCTAACACTGACGT	249
F5	5′-TTCTACTGACTCTAACACTGACGTTGAAGAAACC	243
F6	5′-TCTACTGACTCTAACACTGACGTTGAAGAA	242
F7	5′-ACTGACTCTAACACTGACGTTGAAGAAACCG	239
F8	5′-CTGACTCTAACACTGACGTTGAAGAAACCG	238
F9	5′-GAGATAGTGACCGATACTACTGCTGATCTTGAG	152
F10	5′-AGATAGTGACCGATACTACTGCTGATCTTG	151
F11	5′-AGATAGTGACCGATACTACTGCTGATCTTGA	151
F12	5′-ATAGTGACCGATACTACTGCTGATCTTGAG	149
R	5′-Biotin -GTTCGTGAATAACAATACGCAAACAGGAAG	/
Probe 1	5′-FITC-AAGCGCCAGCAGTGGAGTTAGAAGCGAGCG [THF]TACACCACCAACAGA-DDC	/
Probe 2	5′-FITC-TCAAGTAGAGCCGACTAAAACTCAGCCGAA [THF]CCAGCATCTAACACG-DDC	/
AlgF1	5′-TCAGAGAAAGTTGAGCTAACGATT	568
AlgR1	5′-CATCGTCGCCTGAAGTCGCTGT	
16S rRNA-F	5′-AGAGTTTGATCCTGGCTCAG	/
16S rRNA-R	5′-TACGGTTACCTTGTTACGACTT	

(*F*: forward primer. *R*: reverse primer)

### Screening of primers and probes

The 12 primer pairs composed of the 12 forward primers and the reverse primer were screened for their ability to RPA amplify the *V*. *alginolyticus toxR* gene fragments. The results showed that the primer pair F6/R produced the strongest amplification band on the agarose gel and that the primer pairs F1/R, F2/R and F3/R were obviously stronger than the rest of the primer pairs (Fig. 2b). As the primer pair F3/R produced an obvious primer-dimer band on the gel at a lower position of the lane, F1/R, F2/R and F6/R were selected for further screening.

**Fig. 3:**
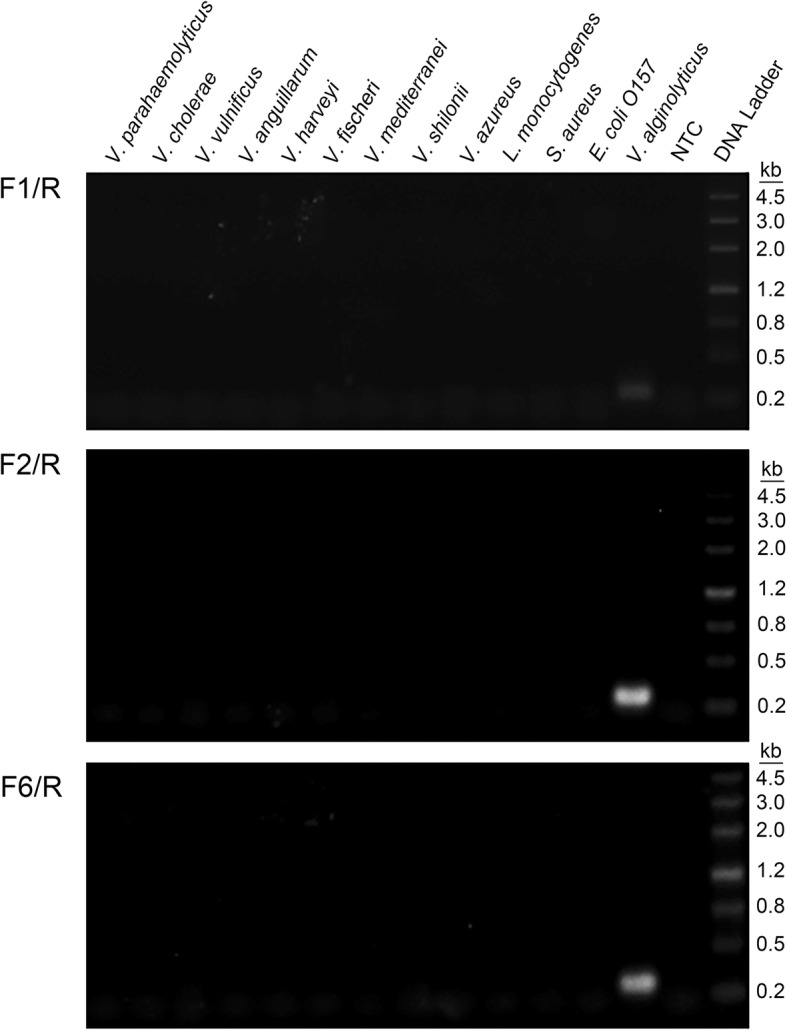
Amplification specificity of the primer pairs. Agarose gel images showing the RPA amplification results for the different bacterial templates by the F1/R, F2/R and F6/R primer pairs. The species of bacteria are indicated on top of each lane. The NTC lane is the no-template control. The size of each band of the DNA ladder is indicated on the right of the gel image

These 3 primer pairs were confirmed for their amplification specificity towards *V*. *alginolyticus* DNA (Fig. 3). As observed on the agarose gel, the 3 primer pairs produced amplification bands only for *V*. *alginolyticus* DNA. No amplification occurred for the other *Vibrio* species or other commonly seen pathogenic bacteria.

**Fig. 4:**
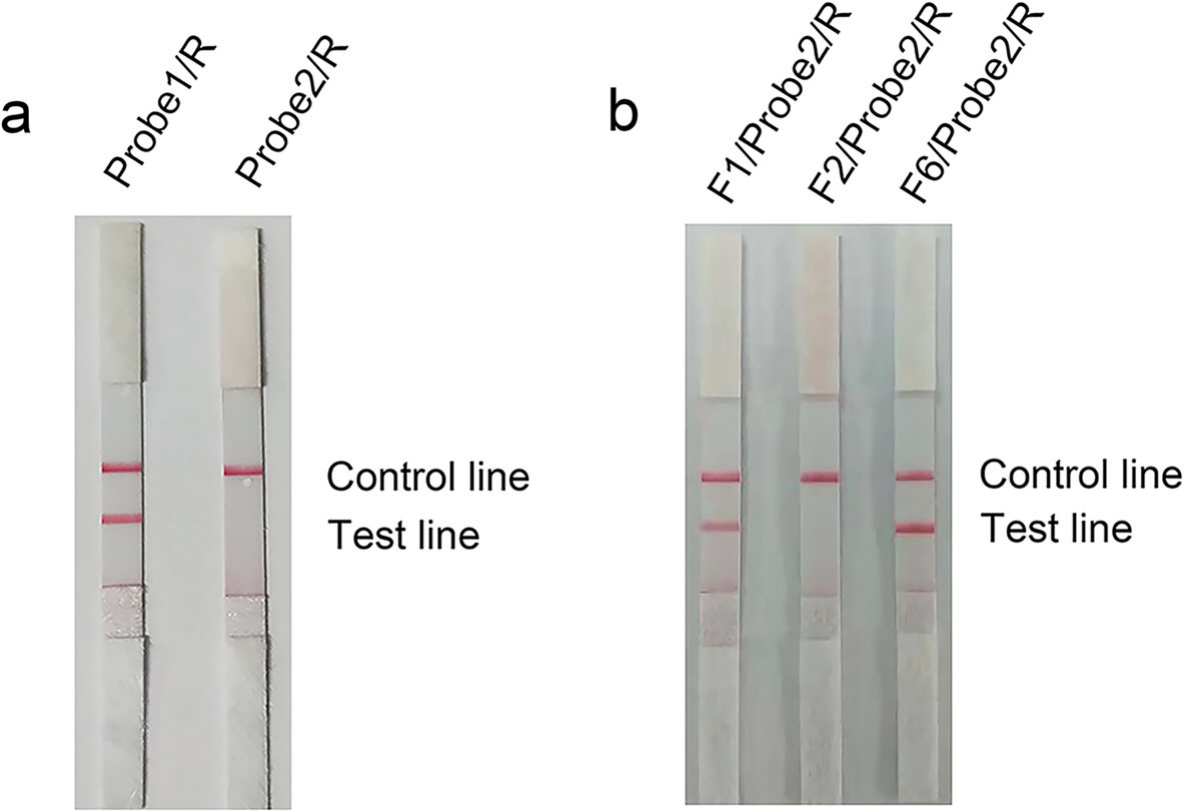
Screening for primer-probe sets without false positive signals. Images of the LFD showing the detection results for the primer-probe sets without any DNA template. The primer-probe set names are indicated on top of each dipstick. The positions of the control and test lines are indicated on the right of the image

According to the pairing positions on the *toxR* gene, both of the probes could work with the 3 primer pairs. To avoid the risk of the probe-primer complex producing a false positive signal on the dipstick without amplification of target DNA, the probes and primers were tested in the RPA reaction without template DNA. With the reverse primer R, Probe 1 but not Probe 2 produced a red band at the test line, which was a false positive signal (Fig. 4a). Then, Probe 2 was tested with the 3 primer pairs, and the results showed that only the primer pair F2/R with Probe 2 did not produce false positive signals (Fig. 4b). Thus, the primer-probe set F2/Probe 2/R was used in the following characterizations of this study.

### Detection specificity of the RPA-LFD method

**Fig. 5:**
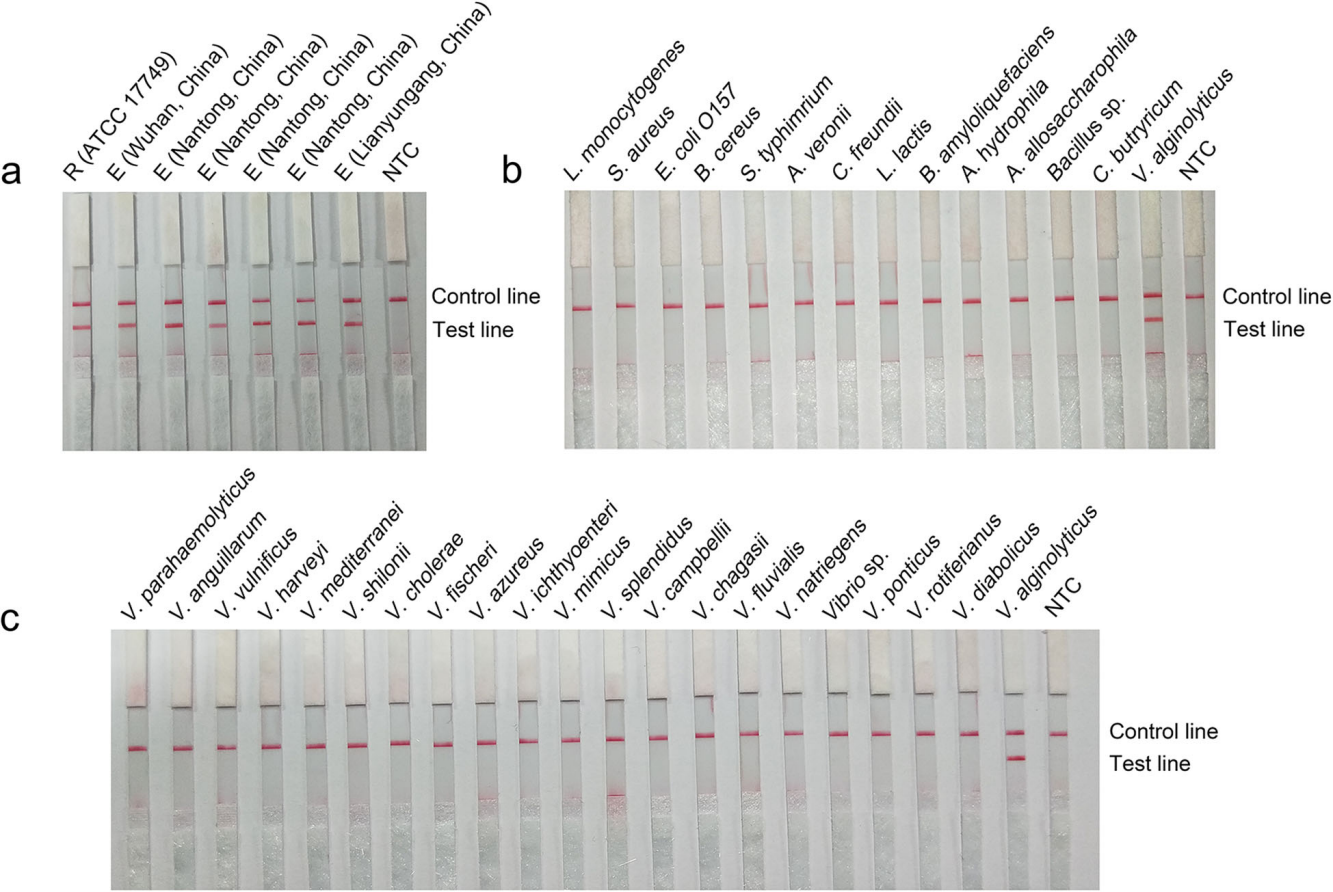
Confirmation of the specificity of the RPA-LFD method. Images of the LFD showing the results of RPA amplification for the different bacterial templates, including the reference (R) *V. alginolyticus* strain and environmental (E) isolations (**a**), other pathogenic bacteria (**b**), and other *Vibrio* species (**c**). The descriptions of the bacteria are indicated on top of each dipstick. The NTC dipstick is the no-template control. The positions of the control and test lines are indicated on the right of the image

The detection specificity of the primer-probe set F2/Probe 2/R in the RPA-LFD method was confirmed using a number of bacterial strains (Table S1). The reference *V*. *alginolyticus* strain and environmental isolates were all detected by this method (Fig. 5a). Other common pathogenic bacteria and *Vibrio* species other than *V*. *alginolyticus* were all negative (Fig. 5, b and c). This result indicated that the RPA-LFD method was highly specific to *V*. *alginolyticus*. Moreover, comparison of the sequences targeted by the probe and primers in the *toxR* gene from *V*. *alginolyticus* with the corresponding sequences of the *toxR* gene from other *Vibrio* species and other bacteria suggested good divergence, which would not allow cross-interaction of the primer-probe set F2/Probe 2/R with other *Vibrio* species or other bacteria (Figure S3).

### Optimization of the RPA-LFD conditions

**Fig. 6:**
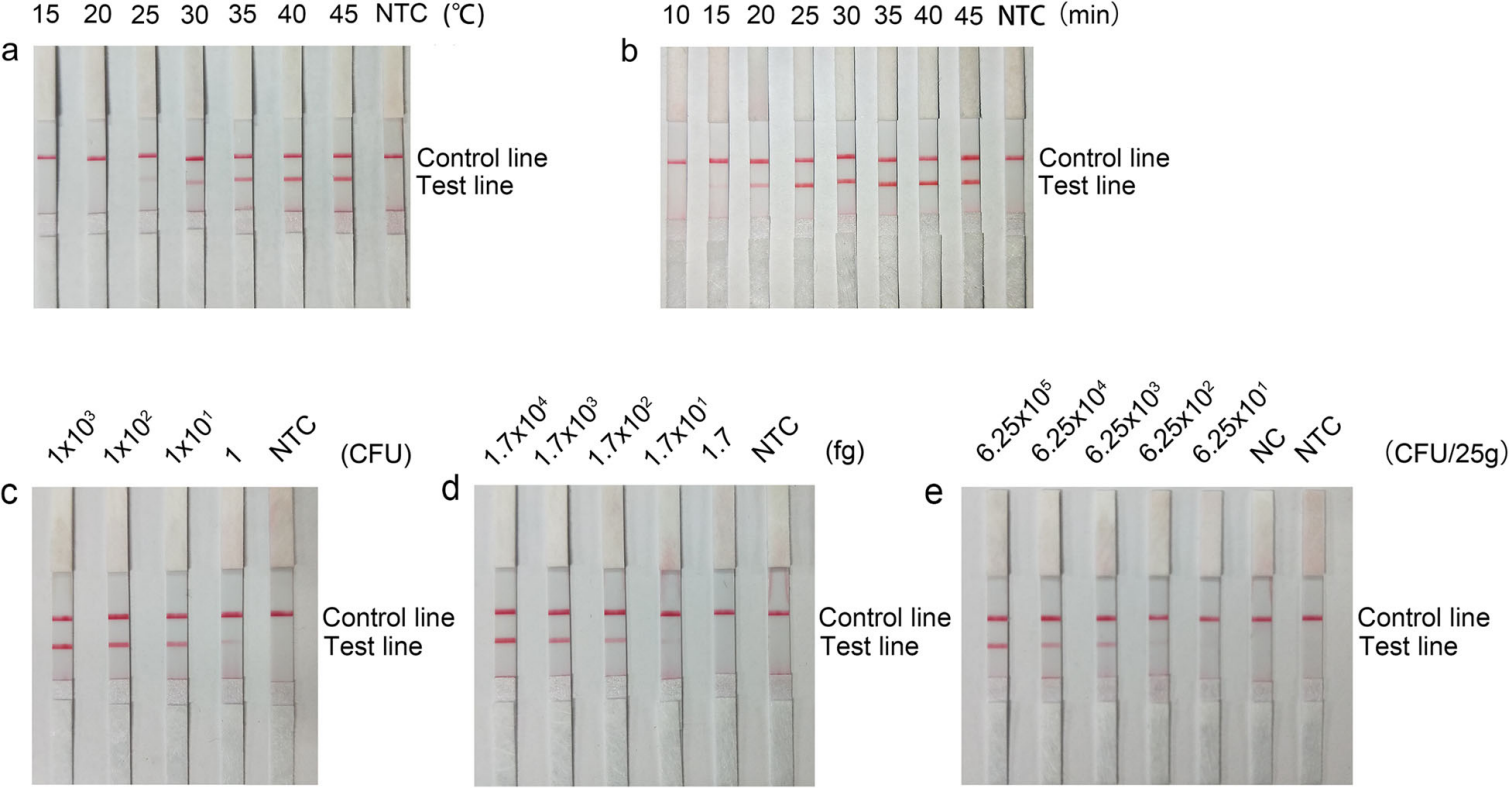
Optimization and detection limit of the RPA-LFD method. (**a**) Image showing the LFD results of RPA amplification under different temperatures. The temperatures under which the RPA reactions were performed are indicated on top of each dipstick. The amplification template was *V*. *alginolyticus*. The NTC dipstick is the no-template control that was performed at 40 °C. (**b**) Image showing the LFD results of RPA amplification with different reaction times. The times for which the RPA reactions were performed are indicated on top of each dipstick. The amplification template was *V*. *alginolyticus*. The NTC dipstick is the no-template control that was performed for 45 min. (**c**) Image showing the LFD results of RPA amplification with different amounts of *V*. *alginolyticus*. The amounts tested are indicated on top of each dipstick. The NTC dipstick is the no-template control. The reactions were performed at 40 °C for 25 min. (**d**) Image showing the LFD results of RPA amplification with different amounts of purified genomic DNA of *V*. *alginolyticus*. The amounts of DNA tested are indicated on top of each dipstick. The NTC dipstick is the no-template control. The reactions were performed at 40 °C for 25 min. (**e**) Image showing the RPA-LFD test results for shrimp samples spiked with *V*. *alginolyticus*. The spiked amounts are indicated on top of each dipstick. The NC dipstick is the control of the shrimp sample without spiking. The NTC dipstick is the no-template control. The reactions were performed at 40 °C for 25 min. The positions of the control and test lines are indicated on the right of the images

The conditions of the RPA reaction were optimized in regard to the reaction temperature and time. RPA amplification of the *V*. *alginolyticus* template was performed under different temperatures ranging from 15 °C to 45 °C (Fig. 6a). The red band at the test line was visible between 25 and 45 °C and achieved the best thickness at 40 °C and 45 °C. At 40 °C, the reaction time was tested from 10 min to 45 min (Fig. 6b). The red band at the test line appeared from 15 min onwards and remained the same thickness from 25 min to 45 min. Therefore, it was determined that the best reaction conditions for RPA-LFD detection were 40 °C for 25 min.

### Detection limit of the RPA-LFD method for *V. alginolyticus*

An inactivated culture of *V*. *alginolyticus* was 10-fold serially diluted, and 1 to 10^3^ colony forming units (CFU) of *V*. *alginolyticus* (in 1 μL for a 50-μL reaction) was tested with the RPA-LFD method. A weak red band at the test line was observed with 1 CFU, and the band thickness increased with increasing quantities of *V*. *alginolyticus* (Fig. 6c). Under similar conditions, 10-fold series dilutions of purified *V*. *alginolyticus* genomic DNA were tested. As low as 170 fg of *V*. *alginolyticus* genomic DNA could be detected (Fig. 6d). To test whether the system could resist interference from factors from real samples, shrimp homogenate samples were spiked with different amounts of *V*. *alginolyticus*. With no enrichment step, DNA was extracted and purified from the spiked samples and tested with the RPA-LFD method. Samples spiked with 6.25 × 10^3^ CFU/25 g or higher showed distinct positive signals (Fig. 6e). We concluded that the detection limit of the RPA-LFD method for *V*. *alginolyticus* was 1 CFU per reaction without DNA purification, or 170 fg of genomic DNA/50 μL, or 6.25 × 10^3^ CFU/25 g in spiked shrimp without any enrichment.

### Application simulation of the RPA-LFD method for *V. alginolyticus* detection

A simulation using the RPA-LFD method for *V*. *alginolyticus* detection in spiked shrimp samples was conducted, and the detection accuracy was compared with that of the traditional PCR method [40]. Fifty-eight shrimp samples were prepared, with 6 spiked with *V*. *alginolyticus*. The 58 samples were randomly numbered and subjected to detection of *V*. *alginolyticus* with both RPA-LFD and PCR. All the spiked samples were successfully detected, and the results of the RPA-LFD method were consistent with the PCR results (Table 2 and Figure S4).

**Table 2:** Detection accuracy of RPA-LFD and PCR for *V*. *alginolyticus*

Sample number	Detection methods		Sample number	Detection methods		Sample number	Detection methods	
	RPA-LFD	PCR			PCR		RPA-LFD	PCR
1	–	–	21	–	–	41	–	–
2	+	+	22	–	–	42	–	–
3	–	–	23	–	–	43	+	+
4	–	–	24	–	–	44	–	–
5	–	–	25	–	–	45	–	–
6	–	–	26	–	–	46	–	–
7	+	+	27	–	–	47	–	–
8	–	–	28	–	–	48	–	–
9	–	–	29	–	–	49	–	–
10	–	–	30	–	–	50	–	–
11	–	–	31	–	–	51	–	–
12	–	–	32	–	–	52	–	–
13	–	–	33	–	–	53	–	–
14	–	–	34	+	+	54	+	+
15	–	–	35	–	–	55	+	+
16	–	–	36	–	–	56	–	–
17	–	–	37	–	–	57	–	–
18	–	–	38	–	–	58	–	–
19	–	–	39	–	–			
20	–	–	40	–	–			

(+: positive result. -: negative result)

## Discussion

Fast and specific detection of *V*. *alginolyticus* pathogenic strains is important in the mariculture industry to control its outbreaks and prevent economic losses [41]. The detection of *V*. *alginolyticus* has to be specific to facilitate pointed treatment and prevention [42]. Rapid and accurate on-site detection is essential because of the fast onset and outbreak of the pathogen [41]. The RPA-LFD combined method is a promising solution because it is fast, simple and can target specific gene sequences [34].

Selection of the detection target was important for specificity. *V. alginolyticus* also includes nonpathogenic strains, which are beneficial for aquatic animals and should not be detected [3]. Virulence genes are usually selected as biomarkers in PCR-based detection. Among them, the *toxR* gene has been reported to cover all the pathogenic strains and was selected as our target in this study [43]. The *toxR* gene exists not only in *V*. *alginolyticus* pathogenic strains but also in other virulent *Vibrio* species. Thus, we had to carefully select regions within this gene that were specific to *V*. *alginolyticus*.

To find regions specific to *V. alginolyticus*, the *toxR* gene sequence of *V. alginolyticus* were compared with those from a series of other *Vibrio* species, and the diverged regions were selected (Figure S1). These regions were then compared within *V*. *alginolyticus* pathogenic strains (Figure S2). Only the areas that diverged in *Vibrio* species and were conserved in *V*. *alginolyticus* pathogenic strains were considered possible targeting areas for the primers and probes in the RPA system. This method ensured that our RPA system to was specific for all *V*. *alginolyticus* pathogenic strains. In the complicated RPA reaction, small variations in the position and length of the primers and probe can affect the amplification result. Thus, multiple primers and probes were designed for systematic screening (Fig. 2a). The screening considered the amplification performance, primer-dimer formation and false positive signals. The amplification specificity was thoroughly investigated for a number of *V*. *alginolyticus* isolates, other *Vibrio* species and other pathogenic bacteria. Finally, a primer-probe set that was highly specific to *V*. *alginolyticus* pathogenic strains was obtained (Fig. 5).

After optimization, our RPA-LFD method was able to detect as little as 1 CFU of *V*. *alginolyticus* per reaction without DNA purification, or 170 fg of genomic DNA (equivalent to 30 copies of *V*. *alginolyticus* genomic DNA) [44], or 6.25 × 10^3^ CFU/25 g in spiked shrimp without any enrichment. This detection limit was satisfactory compared to those of the currently available detection methods for *V*. *alginolyticus*, such as multiplex PCR, loop-mediated isothermal amplification (LAMP) and quantitative PCR. The detection limit of multiplex PCR is 10 CFU or 5 × 10^2^ to 5 × 10^1^ copies of genomic DNA [45]. The detection limit of LAMP is 2 × 10^4^ CFU/g of spiked shrimp [46]. The quantitative PCR method has a better detection limit than those of multiplex PCR and LAMP, at 0.14–0.4 pg of genomic DNA per reaction [47], which is at the same level as our RPA-LFD method. Moreover, the RPA-LFD method was simple and fast. Detection was finished within 30 min at temperatures between 35 °C and 45 °C, and the visual signal on the dipstick could be directly read with the naked eye. In an application simulation, randomly spiked shrimp homogenate samples were 100% accurately detected. Our study provides an RPA-LFD method for *V*. *alginolyticus* detection that is fast, simple, and highly specific with good sensitivity.

## Conclusions

A fast and simple RPA-LFD method to detect *V*. *alginolyticus* pathogenic strains with high specificity was established in this study. By carefully selecting the target gene and the specific amplification regions, the RPA-LFD method was able to accurately identify *V*. *alginolyticus* pathogenic strains but not any other *Vibrio* species or other pathogenic bacteria. The method is well suited for the on-site detection of *V*. *alginolyticus* pathogenic strains in the mariculture industry.

## Methods

### Bacterial strains

*V. alginolyticus*, *Listeria monocytogenes*, *Staphylococcus aureus*, *Escherichia coli* O157, and *Bacillus cereus* at a concentration of 10^6^ colony forming units (CFU)/mL in LB medium were kindly provided by the Wuhan Institute for Food and Cosmetic Control (Wuhan, China). Six environmental isolates of *V. alginolyticus* and other bacterial species including *V. parahaemolyticus*, *V. anguillarum*, *V. vulnificus*, *V. harveyi*, *V. mediterranei*, *V. shilonii*, *V. cholerae*, *V. fischeri*, *V. azureus*, *S. typhimurium*, *Aeromonas veronii*, *Citrobacter freundii*, *Leuconostoc lactis*, *B. amyloliquefaciens*, *A*. *allosaccharophila*, and *Clostridium butyricum* at a concentration of 10^6^ CFU/mL in LB medium were kindly provided by the Jiangsu Institute of Oceanology and Marine Fisheries (Nantong, China). *V*. *ichthyoenteri*, *V*. *mimicus*, *V*. *splendidus*, *V*. *campbellii*, *V*. *chagasii*, *V*. *fluvialis*, *V*. *natriegens*, *Vibrio* sp., *V*. *ponticus*, *V*. *rotiferianus*, and *V*. *diabolicus* at a concentration of 10^6^ CFU/mL in LB medium were kindly provided by the Ministry of Natural Resources Third Institute of Oceanography (Xiamen, China). The details of the strains are listed in Table S1. All the bacterial species were confirmed by 16S rRNA sequencing [48]. Briefly, a single colony of the bacterium was boiled at 100 °C in water for 2 min, and the supernatant containing the 16S rRNA gene was used as the template for PCR amplification with the 16S rRNA general primers (16S rRNA-F and 16S rRNA-R; the sequences are provided in Table 1). The PCR product was sequenced (General Biosystems, Anhui, China) and confirmed to be the corresponding species using NCBI BLAST.

### Sequences and alignments

The nucleic acid sequences of the *toxR* genes were downloaded from GenBank. The alignments were performed with ClustalX software with the default parameter settings [49]. The alignment images were produced with ESPript software (http://espript.ibcp.fr).

### Design of primers and probes

The primers were designed with Primer Premier 5.0 software (Premier Biosoft International, CA, USA). For the primers, after the sequence of a particular targeting region was input, the parameters were set as follows: PCR product size, 100 bp to 300 bp; primer length, 30 bp to 35 bp. For the probes, the parameters were set as follows: PCR product size, 100 bp to 300 bp; primer length, 46 bp to 51 bp. The possibility of pairing between the forward and reverse primers and the probe and the reverse primer was manually checked. Primers with sequence pairing of more than 3 consecutive bases (and more than 1 base if at the 3′ end) were abandoned. The sequences of the primers and probes were then confirmed for species specificity using Primer BLAST on the NCBI website.

### RPA procedure

Bacterial cultures were treated at 100 °C for 10 min before being used as templates. RPA reactions were set up according to the manufacturer’s instructions for the TwistAmp Liquid DNA Amplification Kit (TwistDx, Inc., Maidenhead, UK). The reaction contained 25 μL of 2 × reaction buffer, 5 μL of 10 × basic e-mix, 2.5 μL of 20 × core mix, 2.1 μL of each primer (10 μM), 9.8 μL of distilled water, 1 μL (the concentration was 10^6^ CFU/mL) of the template and a dried enzyme pellet. The primers were commercially synthesized with the 5′ end of the reverse primer labeled with biotin (General Biosystems). To initiate the reaction, 2.5 μL of magnesium acetate (280 mM) was added to the mixture. After brief centrifugation, the reaction mixture was immediately incubated at 37 °C for 30 min. The RPA amplification products were electrophoresed on a 1.5% agarose gel.

### RPA-LFD procedure

Bacterial cultures were treated at 100 °C for 10 min before being used as templates. For reactions using genomic DNA as the template, genomic DNA from 1 mL of the bacterial culture at 10^6^ CFU/mL was purified using the TIANamp Genomic DNA Kit (Tiangen Biotech Co., Ltd., Beijing, China) into a 50 μL volume. One microliter of the purified DNA was used for RPA-LFD. The amount of genomic DNA was determined with a Qubit 4 fluorometer (Thermo Fisher Scientific, Inc., Waltham, MA, USA) as per the manufacturer’s instructions. The reverse primers and probes were modified with biotin and fluorescein isothiocyanate (FITC) at the 5′ ends, respectively (General Biosystems). The 3′ end of each probe was blocked with a dideoxycytidine (DDC), and a “C” base in the middle of the probe that was at least 30 bases away from the 5′ end and 15 bases away from the 3′ end was replaced with a THF group. RPA reactions were set up according to the manufacturer’s instructions for the TwistAmp DNA Amplification nfo Kit (TwistDx). The reaction contained 29.5 μL of rehydration buffer, 2.1 μL of each primer (10 μM), 0.6 μL of probe (10 μM), 12.2 μL of distilled water, 1 μL of template and a dried enzyme pellet. To initiate the reaction, 2.5 μL of magnesium acetate (280 mM) was added into the mixture. After a brief centrifugation, the reaction mixture was immediately incubated at 15–45 °C for 10–45 min. Five microliters of the amplification products were used for LFD (Ustar Biotechnologies Ltd., Hangzhou, China) detection. The amplification products were added to the sample pad of the LFD, and the stick of the LFD was inserted into 100 μL of the sample buffer (Ustar Biotechnologies) for 5 min before visual reading.

### Preparation of spiked shrimp homogenate samples

Shrimp were purchased from a local market and verified to be free of *V. alginolyticus* by PCR [40]. The PCR primers are listed in Table 1. To test the detection limit in the spiked shrimp sample, 1 mL of serially diluted *V. alginolyticus* culture from 2.5 × 10^4^ CFU/mL to 2.5 CFU/mL was mixed with 1 g of shrimp homogenate (shrimp were ground in liquid nitrogen) and 9 mL alkaline peptone broth (Sinopharm Chemical Reagent Co., Ltd., China). Genomic DNA was purified using the TIANamp Genomic DNA Kit (Tiangen Biotech) in a 50 μL volume. One microliter of the purified DNA was used for RPA-LFD. For application simulation, the shrimp was cut into small pieces, and 300 mg of the pieces was ground in liquid nitrogen for each sample. Into several randomly selected homogenate samples, 3 μL of the 10^6^ CFU/mL *V*. *alginolyticus* culture was mixed. DNA was purified from the homogenate samples by the TIANamp Genomic DNA Kit (Tiangen Biotech) in a 50 μL volume. One microliter of the purified DNA was used for RPA-LFD or PCR detection.

### PCR detection of *V. alginolyticus* in shrimp samples

One microliter of purified DNA from the shrimp sample was used as the template for PCR amplification with the primers previously used for *V. alginolyticus* detection [40]. The PCR primers are listed in Table 1. Five microliters of the PCR product was electrophoresed on a 1% agarose gel.

## Supplementary information

**Additional file 1: Fig. S1** Sequence alignment of the *toxR* genes of *Vibrio* species. The name of the corresponding species is indicated on the left of each sequence. GenBank numbers of the *toxR* genes of the species are EU155543.1, DQ640258.1, AB175481.1, EU727207.1, EU727208.1, AB029907.1, MF100077.1, and AB042547.1 (from top to bottom). Diverged regions are indicated by the black boxes. **Fig. S2** Sequence alignment of the *toxR* genes of *V*. *alginolyticus* pathogenic strains. Information of the corresponding strain is indicated on the left of each sequence. GenBank numbers of the *toxR* genes of the strains are EU155543.1, CP014036.1, AB372531.1, AB372526.1, CP014036.1, and JN188451.1 (from top to bottom). The conserved region is indicated by the black box. **Fig. S3.** Sequences targeted by primer-probe set F2/Probe 2/R in the *toxR* genes of *Vibrio* species and other bacteria. The name of the corresponding species is indicated on the left of each sequence. GenBank numbers of the *toxR* genes of the species are EU155543.1, HQ452616.1, LT797832.1, NC_002516.2, NC_012214.1, AY751345.1, JX401922.1, HQ318823.1, KU760757.1, KX280762.1, AF414370.1, MF100077.1, NC_006370.1, AM183574.1, and AB042547.1 (from top to bottom). The targeted regions by the forward primer (F), the probe (P) and the reverse primer (R) are indicated by the black boxes. Sequences targeted by primer-probe set F2/Probe 2/R in the *toxR* genes of *Vibrio* species and other bacteria. GenBank numbers of the *toxR* genes of the species are EU155543.1, KT265743.1, HQ452618.1, EU727207.1, AY751346.1, GQ455024.1, AY751359.1, AY751341.1, AY751340.1, AY751337.1, L29053.1, AY247418.1, AF170885.1, UHIH01000001.1, and AF170884.1 (from top to bottom). Sequences targeted by primer-probe set F2/Probe 2/R in the *toxR* genes of *Vibrio* species and other bacteria. GenBank numbers of the *toxR* genes of the species are EU155543.1, EU155587.1, NC_016613.1, FM999823.1, AY751344.1, AY751342.1, AY751343.1, AF170881.1, EU727208.1, KF322110.1, and HQ452617.1 (from top to bottom). **Fig. S4.** Agarose gel image of the PCR detection of *V. alginolyticus* in shrimp samples. Sample numbers are indicated on the wells of the gel. The DNA ladder was run in the last lane of each row. The band sizes of the DNA ladder are shown on the right of each row. **Table S1.** Strain information of bacteria used in this study and RPA-LFD detection results.

## Data Availability

The datasets analyzed during the current study are available in the NCBI GenBank repository. Each GenBank number was listed in the figure captions of Figure S1, Figure S2, and Figure S3 (Supplementary file).
