# Supplementary material for: Fast, simple and highly specific molecular detection of Vibrio alginolyticus pathogenic strains using a visualized isothermal amplification method
Source: BMC Vet Res. 2020 Mar 4;16:76. doi: 10.1186/s12917-020-02297-4 (PMC7057676; doi:10.1186/s12917-020-02297-4)
Supplement: Supplementary file 1 — Additional file 1: Fig. S1 Sequence alignment of the toxR genes of Vibrio species. The name of the corresponding species is indicated on the left of each sequence. GenBank numbers of the toxR genes of the species are EU155543.1, DQ640258.1, AB175481.1, EU727207.1, EU727208.1, AB029907.1, MF100077.1, and AB042547.1 (from top to bottom). Diverged regions are indicated by the black boxes. Fig. S2 Sequence alignment of the toxR genes of V. alginolyticus pathogenic strains. Information of the corresponding strain is indicated on the left of each sequence. GenBank numbers of the toxR genes of the strains are EU155543.1, CP014036.1, AB372531.1, AB372526.1, CP014036.1, and JN188451.1 (from top to bottom). The conserved region is indicated by the black box. Fig. S3. Sequences targeted by primer-probe set F2/Probe 2/R in the toxR genes of Vibrio species and other bacteria. The name of the corresponding species is indicated on the left of each sequence. GenBank numbers of the toxR genes of the species are EU155543.1, HQ452616.1, LT797832.1, NC_002516.2, NC_012214.1, AY751345.1, JX401922.1, HQ318823.1, KU760757.1, KX280762.1, AF414370.1, MF100077.1, NC_006370.1, AM183574.1, and AB042547.1 (from top to bottom). The targeted regions by the forward primer (F), the probe (P) and the reverse primer (R) are indicated by the black boxes. Sequences targeted by primer-probe set F2/Probe 2/R in the toxR genes of Vibrio species and other bacteria. GenBank numbers of the toxR genes of the species are EU155543.1, KT265743.1, HQ452618.1, EU727207.1, AY751346.1, GQ455024.1, AY751359.1, AY751341.1, AY751340.1, AY751337.1, L29053.1, AY247418.1, AF170885.1, UHIH01000001.1, and AF170884.1 (from top to bottom). Sequences targeted by primer-probe set F2/Probe 2/R in the toxR genes of Vibrio species and other bacteria. GenBank numbers of the toxR genes of the species are EU155543.1, EU155587.1, NC_016613.1, FM999823.1, AY751344.1, AY751342.1, AY751343.1, AF170881.1, EU727208.1, KF322110.1, and HQ452 [file 12917_2020_2297_MOESM1_ESM.pdf]

Supplementary Information

Fast, simple and highly specific molecular detection of *Vibrio alginolyticus* pathogenic strains using a visualized isothermal amplification method

Yu Dong[1,†], Panpan Zhao[2,†], Li Chen[1], Huahua Wu[1], Xinxin Si[1], Xin Shen[1], Hui Shen[3], Yi Qiao[3], Shanyuan Zhu[4], Qiong Chen[5], Weiwei Jia[1], Jingquan Dong[1,*], Juan Li[5,*], Song Gao[6,*]

[1]Jiangsu Key Laboratory of Marine Biological Resources and Environment, Jiangsu Key Laboratory of Marine Pharmaceutical Compound Screening, Co-Innovation Center of Jiangsu Marine Bio-industry Technology, Jiangsu Ocean University, Lianyungang 222005, China.

[2]Key Laboratory of Zoonosis Research by Ministry of Education, College of Veterinary Medicine, Jilin University, Changchun 130062, China.

[3]Jiangsu Institute of Oceanology and Marine Fisheries, Nantong 226007, China.

[4]Jiangsu Agri-animal Husbandry Vocational College, Taizhou 225300, China.

[5]Wuhan Institute for Food and Cosmetic Control, Wuhan 430000, China.

[6]School of Pharmacy, Jiangsu Ocean University, Lianyungang 222005, China.

[†]These authors contributed equally to this paper.

[*]Correspondence Email addresses: 2018000029@jou.edu.cn (J.D.) / lijuanice81@163.com (J.L.) / gaos@jou.edu.cn (S.G.)

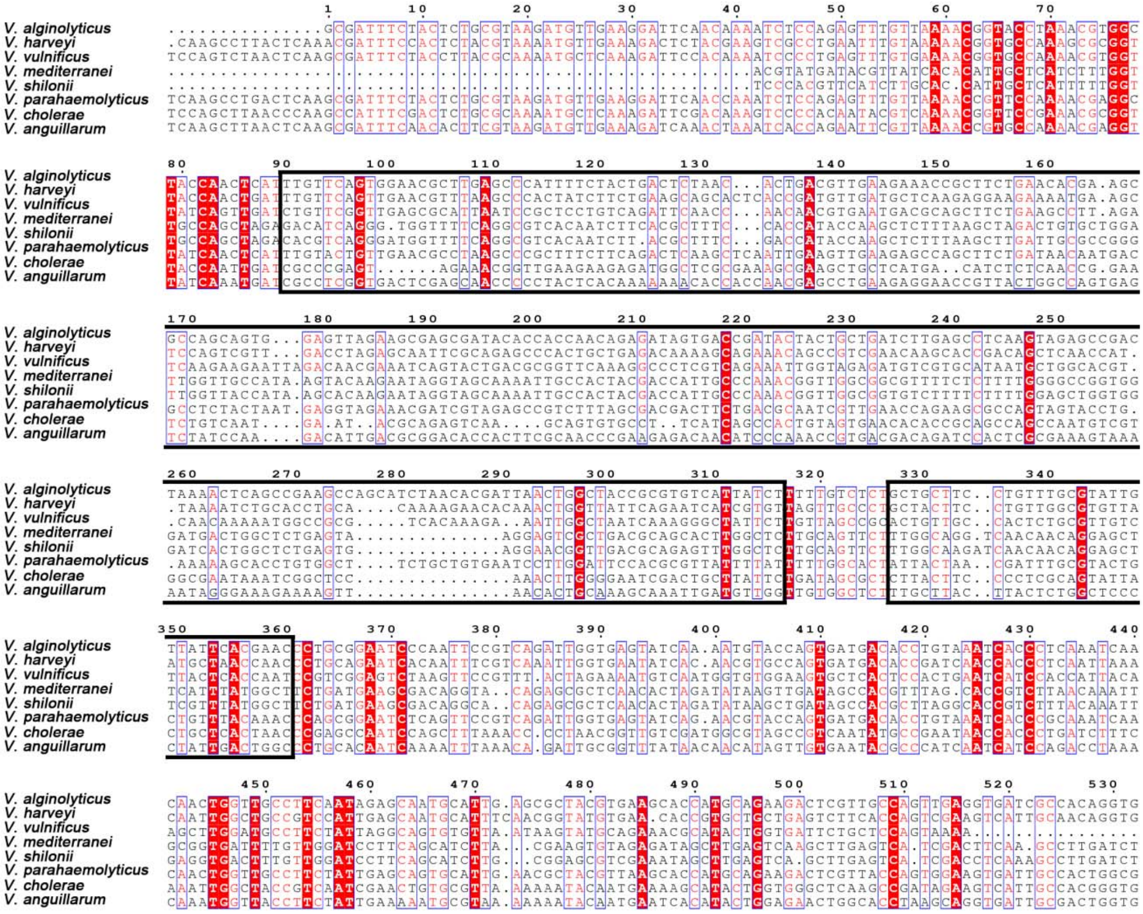

Fig. S1 Sequence alignment of the *toxR* genes of *Vibrio* species. The name of the corresponding species is indicated on the left of each sequence. GenBank numbers of the *toxR* genes of the species are EU155543.1, DQ640258.1, AB175481.1, EU727207.1, EU727208.1, AB029907.1, MF100077.1, and AB042547.1 (from top to bottom). Diverged regions are indicated by the black boxes.

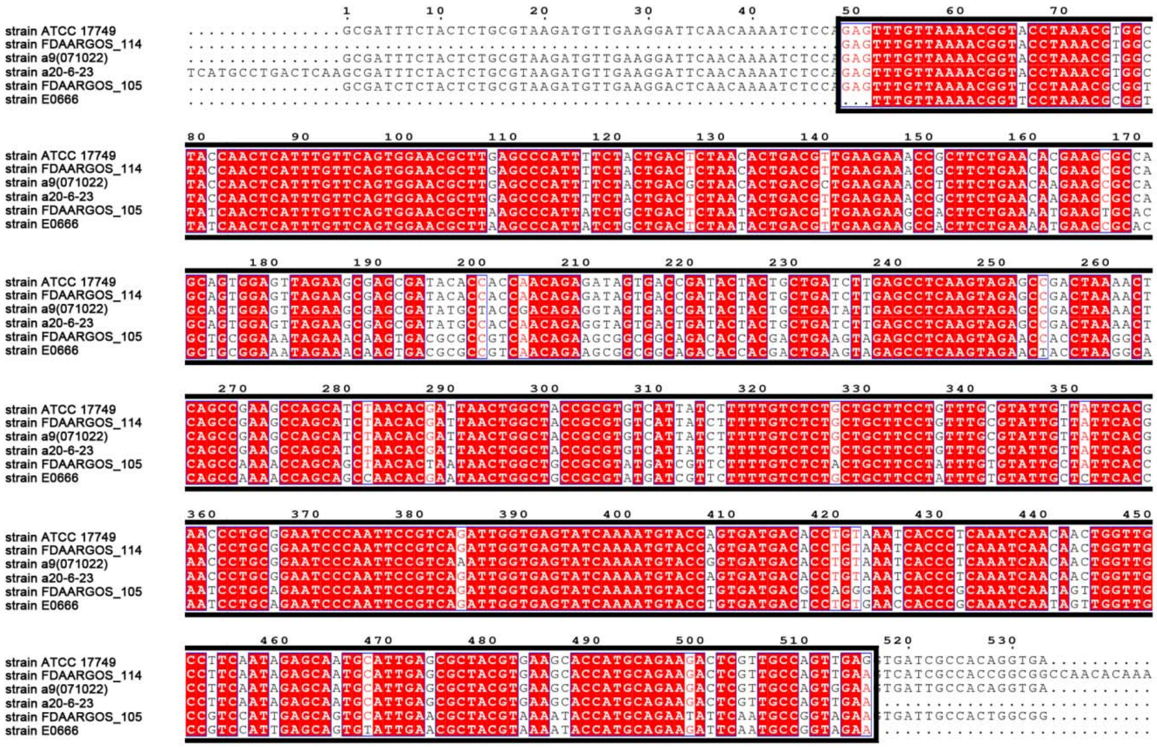

Fig. S2 Sequence alignment of the *toxR* genes of *V. alginolyticus* pathogenic strains. Information of the corresponding strain is indicated on the left of each sequence. GenBank numbers of the *toxR* genes of the strains are EU155543.1, CP014036.1, AB372531.1, AB372526.1, CP014036.1, and JN188451.1 (from top to bottom). The conserved region is indicated by the black box.

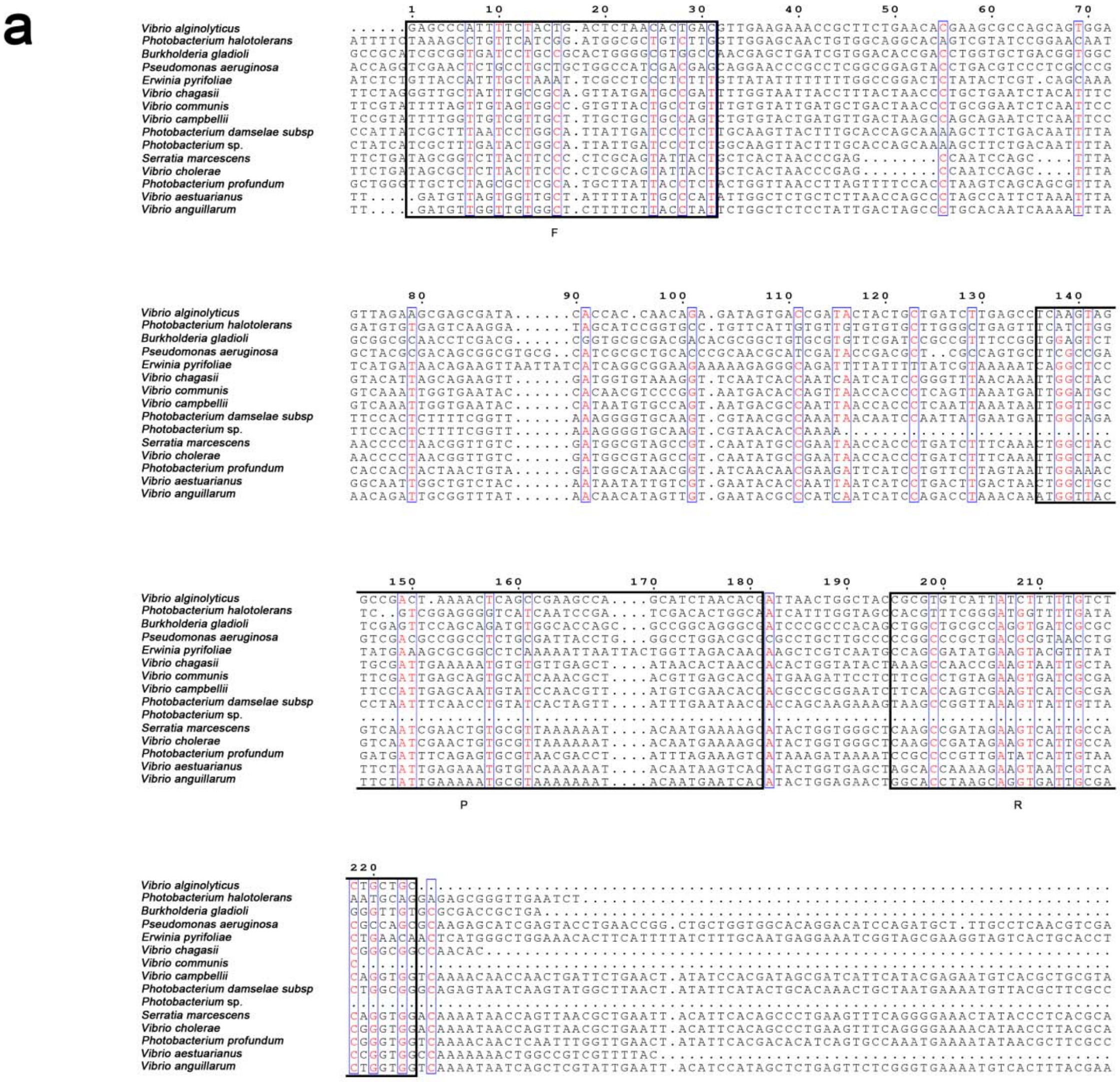

Fig. S3 Sequences targeted by primer-probe set F2/Probe 2/R in the *toxR* genes of *Vibrio* species and other bacteria. The name of the corresponding species is indicated on the left of each sequence. GenBank numbers of the *toxR* genes of the species are EU155543.1, HQ452616.1, LT797832.1, NC_002516.2, NC_012214.1, AY751345.1, JX401922.1, HQ318823.1, KU760757.1, KX280762.1, AF414370.1, MF100077.1, NC_006370.1, AM183574.1, and AB042547.1 (from top to bottom). The targeted regions by the forward primer (F), the probe (P) and the reverse primer (R) are indicated by the black boxes.

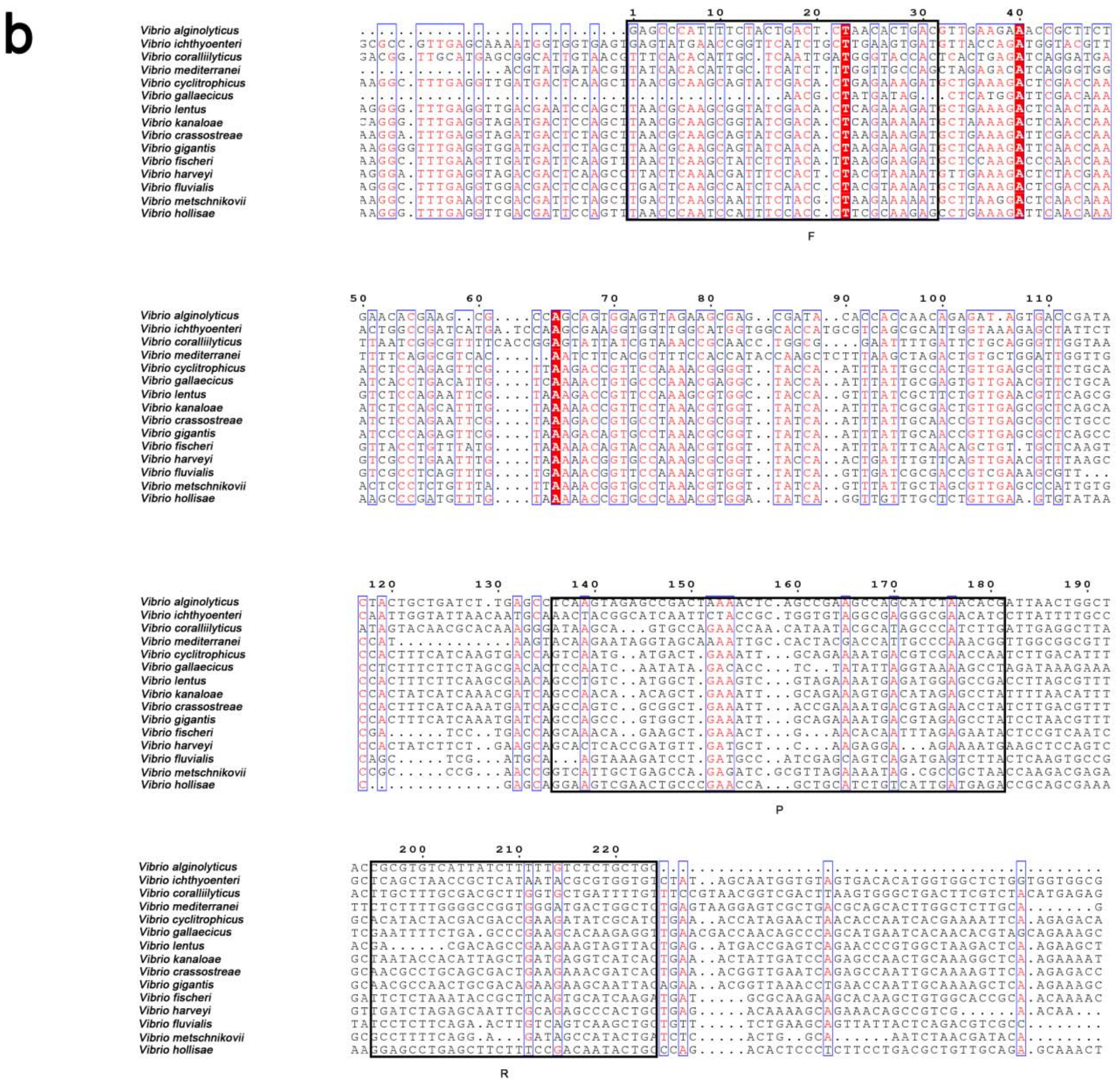

Fig. S3 (continued) Sequences targeted by primer-probe set F2/Probe 2/R in the *toxR* genes of *Vibrio* species and other bacteria. GenBank numbers of the *toxR* genes of the species are EU155543.1, KT265743.1, HQ452618.1, EU727207.1, AY751346.1, GQ455024.1, AY751359.1, AY751341.1, AY751340.1, AY751337.1, L29053.1, AY247418.1, AF170885.1, UHIH01000001.1, and AF170884.1 (from top to bottom).

C

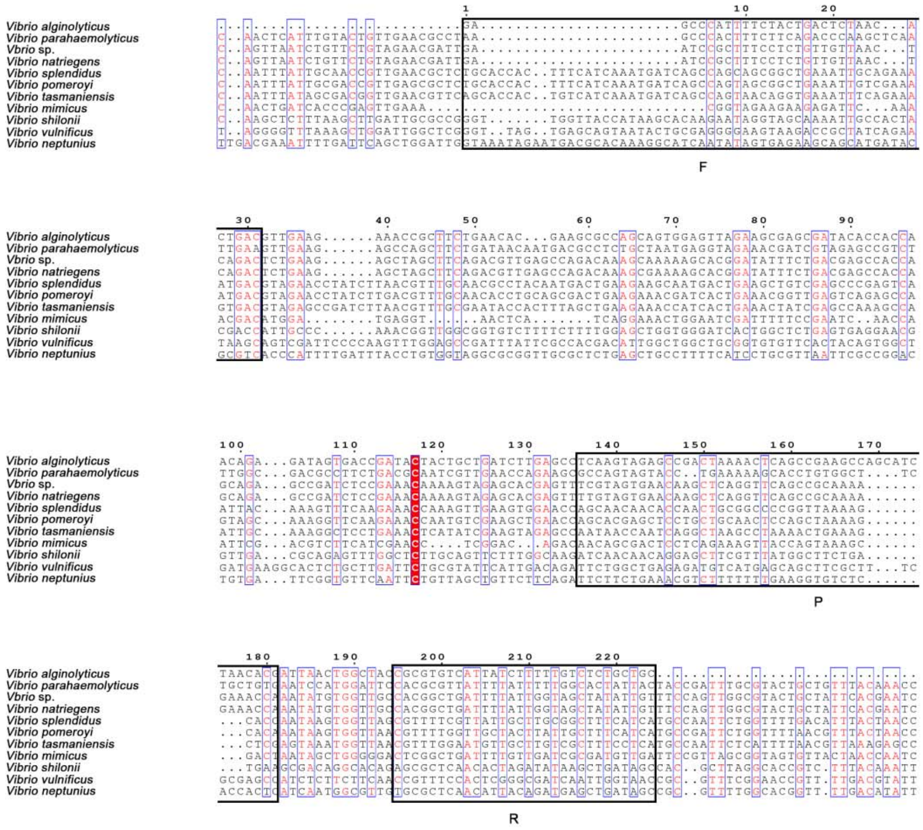

Fig. S3 (continued) Sequences targeted by primer-probe set F2/Probe 2/R in the *toxR* genes of *Vibrio* species and other bacteria. GenBank numbers of the *toxR* genes of the species are EU155543.1, EU155587.1, NC_016613.1, FM999823.1, AY751344.1, AY751342.1, AY751343.1, AF170881.1, EU727208.1, KF322110.1, and HQ452617.1 (from top to bottom).

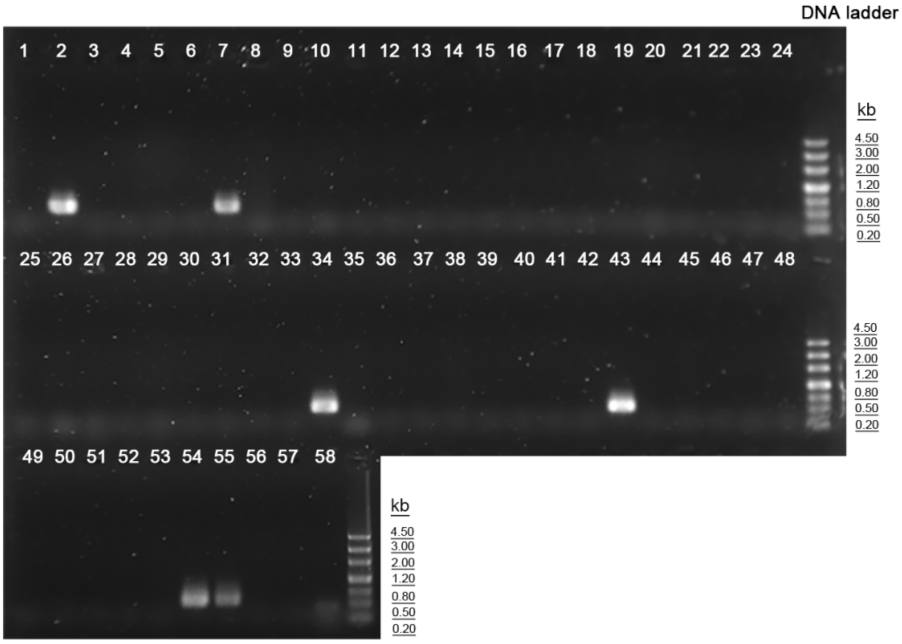

Fig. S4 Agarose gel image of the PCR detection of *V. alginolyticus* in shrimp samples. Sample numbers are indicated on the wells of the gel. The DNA ladder was run in the last lane of each row. The band sizes of the DNA ladder are shown on the right of each row.

Table S1 Strain information of bacteria used in this study and RPA-LFD detection results

Species	Source	strain	RPA-LFD detection results
Vibrio alginolyticus	Reference strain	ATCC 17749	+ (positive)
V. alginolyticus	Environmental strain	Nantong, China	+
V. alginolyticus	Environmental strain	Nantong, China	+
V. alginolyticus	Environmental strain	Nantong, China	+
V. alginolyticus	Environmental strain	Nantong, China	+
V. alginolyticus	Environmental strain	Nantong, China	+
V. alginolyticus	Environmental strain	Lianyungang, China	+
V. parahaemolyticus	Reference strain	ATCC 17802	- (negative)
V. anguillarum	Reference strain	ATCC 19264	-
V. vulnificus	Reference strain	ATCC 27562	-
V. harveyi	Reference strain	ATCC 43516	-
V. mediterranei	Reference strain	ATCC 43341	-
V. shilonii	Reference strain	ATCC BAA-91	-
V. cholera	Reference strain	ATCC 14100	-
V. fischeri	Reference strain	ATCC 700601D	-
V. azureus	Reference strain	MCCC 1A06651	-
V. ichthyoenteri	Reference strain	MCCC 1A00057	-
V. mimicus	Reference strain	MCCC 1A02602	-
V. splendidus	Reference strain	MCCC 1A04096	-
V. campbellii	Reference strain	MCCC 1A02605	-
V. chagasii	Reference strain	MCCC 1B00386	-
V. fluvialis	Reference strain	MCCC 1A02761	-
V. natriegens	Reference strain	MCCC 1D00129	-
Vibrio sp.	Reference strain	MCCC 1A00047	-
V. ponticus	Reference strain	MCCC 1H00061	-
V. rotiferianus	Reference strain	MCCC 1B00068	-
V. diabolicus	Reference strain	MCCC 1D00126	-
Listeria monocytogenes	Reference strain	ATCC 19115	-
Staphylococcus aureus	Reference strain	ATCC 6538	-
Escherichia coli O157:H7	Reference strain	ATCC 43888	-
Bacillus cereus	Reference strain	ATCC 14579	-
S. typhimurium	Reference strain	ATCC 14028	-
B. subtilis	Reference strain	ATCC 6051	-
Aeromonas veronii	Reference strain	MCCC 1A00180	-
Citrobacter freundii	Reference strain	MCCC 1A00198	-
Leuconostoc lactis	Reference strain	MCCC 1A07814	-
B. amyloliquefaciens	Reference strain	ATCC 23842	-
A. hydrophila	Reference strain	MCCC 1A00007	-
A. allosaccharophila	Reference strain	ATCC 51208	-
Bacillus sp.	Reference strain	MCCC 1A00006	-
Clostridium butyricum	Reference strain	ATCC 3627	-
